# Tau pathology increases α‐synuclein spreading from the periphery to the CNS

**DOI:** 10.1002/alz.090546

**Published:** 2025-01-03

**Authors:** Carlos M Soto‐Faguás, Anna Bowman, Randall Woltjer, Vivek K Unni

**Affiliations:** ^1^ Oregon Health & Science University, Portland, OR USA; ^2^ NIA‐Layton Oregon Alzheimer’s Disease Research Center, Oregon Health & Science University, Portland, OR USA

## Abstract

**Background:**

Dementia with Lewy Bodies (DLB) is one of the most common Alzheimer’s Disease (AD)‐related dementias and it is defined by the presence of abnormal cytoplasmic inclusions composed of aggregated α‐synuclein (αsyn) in neuronal soma, known as Lewy bodies (LB). LB often coexists with AD type pathology such as amyloid‐β (Aβ) plaques and neurofibrillary tangles containing hyperphosphorylated tau in several LB dementias, including Parkinson’s Disease Dementia and Lewy Body variant AD. These co‐pathologies likely represent a spectrum of various contributions of shared mechanisms that underlie these diseases. However, the mechanisms of the interaction of these pathological proteins is still poorly understood. Furthermore, there is a lack of mammalian model systems demonstrating robust generation of both Lewy with Aβ and/or tau pathology in the field. Unraveling the mechanistic link between αsyn and AD‐related pathologies might be useful for the treatment of DLB.

**Method:**

We have recently developed a brand‐new transgenic mouse line that expresses A53T αsyn tagged with GFP (A53T Syn‐GFP, AA) that forms LB pathology ∼10x faster than previous models after intracortical/peripheral PFF injection. To investigate the role of tau in αsyn spreading from the periphery to the CNS we crossed the AA mice with the P301S tau transgenic mice (PS19). We have performed monomeric/PPF injection in the gastrocnemius muscle of single and double transgenic mice and carried out a histological analysis of the αsyn and tau pathologies through the spinal cord and the brain.

**Result:**

We found an increase in the number of αsyn+ inclusion induced by PFF injection in the spinal cord and the brain of PS19/AA mice compared to single AA mice. Interestingly, we found sex differences in αsyn pathology being higher in females than males. Furthermore, monomeric αsyn caused reduced but some αsyn inclusions in the spinal cord after muscle injection.

**Conclusion:**

Our preliminary data shows an increase in αsyn pathology and spreading from the periphery to the brain in the presence of tau pathology suggesting a link between αsyn and tau pathologies that could represent potential treatment target for the treatment of DLB patients.

